# Interventional pain management in dogs with lumbosacral stenosis: preliminary long-term clinical outcomes of combined foraminal and epidural injections with or without pulsed radiofrequency

**DOI:** 10.3389/fvets.2025.1730491

**Published:** 2026-01-12

**Authors:** Roger Medina-Serra, Francisco G. Laredo, Francesca de Strobel, Sandra Sanchis-Mora, Eliseo Belda

**Affiliations:** 1 Anaesthesia and Pain Management, North Downs Specialist Referrals, Part of Linnaeus Veterinary Limited, Bletchingley, United Kingdom; 2 Escuela Internacional de Doctorado de la Universidad de Murcia, Programa en Ciencias Veterinarias, Universidad de Murcia, Murcia, Spain; 3 Departamento de Medicina y Cirugía Animal, Facultad de Veterinaria, Universidad de Murcia, Murcia, Spain; 4 Hospital Veterinario Universidad de Murcia, Murcia, Spain; 5 Neurology and Neurosurgery, North Downs Specialist Referrals, part of Linnaeus Veterinary Limited, Bletchingley, United Kingdom; 6 Blaise Veterinary Referral Hospital, Birmingham, United Kingdom

**Keywords:** dog, fluoroscopy guidance, foraminal injections, interventional pain management, lumbosacral pain, transforaminal epidural, ultrasound guidance

## Abstract

**Introduction:**

Lumbosacral stenosis is a recognised cause of pain in dogs, often involving disc herniation and foraminal narrowing with associated radiculopathy. In humans, transforaminal injections demonstrate superior outcomes to interlaminar approaches and are frequently combined with pulsed radiofrequency (PRF) at the dorsal root ganglion (DRG) to enhance pain relief. However, their clinical utility in dogs with naturally occurring lumbosacral pain remains unreported.

**Methods:**

This retrospective cohort study evaluated long-term outcomes (capped at 24 months) following a single procedure involving ultrasound- and fluoroscopy-guided foraminal and epidural corticosteroid and local anaesthetic injections, with (PRF; 9 dogs) or without PRF (No-PRF; 9 dogs) at the L7 DRG, in dogs with chronic lumbosacral pain. Outcome measures included clinician-based Lumbosacral Pain (LSPain) Scale scores and caregiver-reported Canine Brief Pain Inventory (CBPI) scores and quality of life (QoL). Clinically relevant improvement was defined as a two-grade reduction in LSPain Scale score and a CBPI decrease of ≥1 point in pain severity and ≥2 points in pain interference. Data were analysed using Fisher’s Exact Test, Wilcoxon signed-rank tests, Mann–Whitney U tests, and generalised linear mixed models in R.

**Results:**

Baseline outcomes did not differ significantly between groups. Pain severity and QoL improved significantly over time within both groups (*p* < 0.05). Dogs receiving PRF had significantly greater QoL improvement (*p* = 0.0247). Clinically relevant improvement was achieved in 9/9 of dogs in the PRF group and 5/9 in the No-PRF group. The median duration of clinically relevant improvement was longer in the PRF group [16.4 (2.2–24) months] than in the No-PRF group [8.9 (0 to 24) months], although this difference did not reach statistical significance in the sample size studied.

**Discussion:**

These preliminary findings suggest that image-guided targeted injections, with or without adjunctive PRF, may provide long-term pain alleviation in dogs with lumbosacral stenosis.

## 1 Introduction

Lumbosacral stenosis is a recognised cause of lumbosacral pain in both human and veterinary patients (1, 2). In dogs, this condition, broadly referred to as Canine Degenerative Lumbosacral Stenosis (CDLSS), is characterised by a range of degenerative changes that frequently coexist at the lumbosacral junction. These typically include intervertebral disc disease (IVDD), hypertrophy of the interarcuate ligament, and facet joint degeneration, ultimately resulting in varying degrees of foraminal and vertebral canal stenosis and neural insult (2–4).

A recent multivariable analysis of a large cohort of dogs undergoing magnetic resonance imaging (MRI) identified lumbosacral radiculopathy as the strongest predictor of lumbosacral pain (5). The same study also demonstrated significant associations between intervertebral disc herniation and foraminal stenosis and the presence of pain. In contrast, other pathologies such as cauda equina compression and facet joint degeneration were not associated with pain. Consistent with earlier reports (2, 6, 7), this study also highlighted the diagnostic challenge posed by overlapping clinical signs between hip and lumbosacral pathology, two conditions with high prevalence in the canine population.

Current management for CDLSS favour conservative treatment, including pharmacological management, physical rehabilitation, lifestyle modification, and epidural corticosteroid injections (7). Surgical options, such as foraminotomy, dorsal laminectomy and discectomy, and lumbosacral distraction and stabilization, are reserved for cases that do not respond to conservative treatment or in patients with significant neurological deficits, and outcomes vary according to case selection and technique employed (7).

Given the multifactorial nature of canine lumbosacral pain and the limitations of correlating imaging findings with clinical signs, there is increasing interest in minimally invasive, image-guided interventional procedures that may serve both diagnostic and therapeutic purposes.

Interlaminar epidural corticosteroid injections have shown favourable short- to mid-term outcomes in dogs with pain associated with lumbosacral stenosis (3, 8). In medicine, transforaminal epidural injections have demonstrated superior therapeutic efficacy to interlaminar approaches in patients with lumbosacral intervertebral disc herniation and radiculopathy, due to their ability to deliver medication directly to the affected nerve root within the ventral epidural space (9). Given that these two conditions have recently been identified as major predictors of lumbosacral pain in dogs, the potential translational benefits of the transforaminal approach merit investigation in the veterinary setting. Transforaminal epidurals are traditionally performed under fluoroscopic or CT guidance (10–12), although ultrasound-guided techniques have more recently been introduced to minimise radiation exposure and reduce the risk of iatrogenic injury to neural structures or inadvertent vascular puncture (13–15). CT-guided transforaminal lumbar injections were first described approximately a decade ago in canine cadavers and clinically healthy dogs without lumbosacral pain (16–18). However, their clinical efficacy in dogs with naturally occurring lumbosacral pathology and associated pain has not been previously reported. In healthy dogs, minor and self-limiting complications such as transient hyperthermia, vascular puncture, and mild reflex changes affecting the pelvic limb were observed post-injection (17). All adverse effects resolved without intervention, and no abnormalities were detected at the end of the study period.

More recently, a combined ultrasound- and fluoroscopy-guided technique has been developed to deliver pulsed radiofrequency (PRF) to the L7 dorsal root ganglion (DRG) and to perform foraminal injections in dogs (19, 20). PRF targeting the DRG exerts its effects through multiple mechanisms, including modulation of ion channel activity, reduction in excitatory neurotransmitter release, inhibition of pro-inflammatory cytokine release, and alteration of intracellular pathways involved in neuroplasticity and inflammation (21–24). These effects collectively decrease neuronal excitability and induce long-term synaptic depression, contributing to sustained pain relief. Evidence supporting the safety and efficacy of DRG-targeted PRF for lumbar radicular pain in medicine is increasing, with studies demonstrating its potential as a standalone therapy or in combination with transforaminal epidural steroid injections to enhance therapeutic outcomes (25–29).

This study aimed to evaluate long-term outcomes in dogs with spinal pain associated with lumbosacral stenosis treated with a single image-guided interventional procedure comprising two separate injections (foraminal and via sacrococcygeal catheter) performed with or without PRF targeting the DRG. The primary objective was to assess clinical outcomes following this intervention, focusing on changes in pain severity and quality of life (QoL) as measured by clinician- and caregiver-based assessments. The secondary objective was to compare these outcomes between dogs treated with injections alone and those receiving additional PRF. We hypothesised that both approaches would result in sustained clinical improvement, with superior outcomes in the PRF group.

## 2 Materials and methods

This observational, retrospective cohort study received ethical approval from the Royal College of Veterinary surgeons (RCVS) Ethics Review Panel (ERP) (2024-078-Serra). Owners provided informed written consent (Appendix 1) for the use of anonymised clinical data for clinical research and publication purposes.

### 2.1 Case selection

Medical records were reviewed for dogs undergoing interventional pain management targeting the lumbosacral spine at North Downs Specialist Referrals between November 2020 and April 2025. Reviewed data included patient signalment, MRI-based diagnoses, clinical examination findings, medications, caregiver-completed questionnaires, and procedural details.

Dogs were included if they had a history of lumbosacral pain lasting 6 weeks or longer, unresponsive to rest and pharmacological treatment, and MRI findings consistent with lumbosacral stenosis. All dogs received ultrasound- and fluoroscopy-guided foraminal and epidural injections, with or without pulsed radiofrequency (PRF). To evaluate the duration of clinical improvement, only dogs with complete follow-up were eligible. For dogs in which pain relief was maintained, a minimum follow-up period of 24 months was required. For dogs in which pain recurred, follow-up was concluded at the time of recurrence, which marked the endpoint for analysis. Transient, self-limiting (not requiring additional medication) pain episodes lasting fewer than 5 days, following a clear traumatic event (e.g., a fall during a walk), deemed unrelated to the underlying lumbosacral pathology, were not classified as recurrence events.

Exclusion criteria included a Canine Osteoarthritis Staging Tool (COASTeR) (30) score ≥3 affecting any joint; anatomical anomalies/variations of the lumbar or sacral spine (e.g., transitional vertebrae); urinary or faecal incontinence; neoplastic disease; or systemic inflammatory disorders, such as infectious or immune mediated conditions, that may affect mobility and contribute to generalised pain states (excluding mild osteoarthritis). Additional exclusion criteria included inconsistent follow-up reporting (e.g., different caregivers reporting CBPI scores at different time points); additional spinal interventions beyond the lumbosacral region; or any surgical procedure during the assessment period. All dogs meeting the inclusion criteria and did not present exclusion criteria were included in the analysis, irrespective of outcome, to avoid bias related to selective follow-up.

### 2.2 Interventional procedures

All procedures were performed under general anaesthesia by the same operator (RMS). With the dog in sternal recumbency and pelvic limbs pulled forward, the lumbosacral region was surgically prepared. Needle positioning for all foraminal procedures was performed using an ultrasound- and fluoroscopy-guided technique originally described for DRG-targeted PRF (20) and subsequently applied for foraminal injections (19).

In dogs not receiving PRF, the foraminal injections were performed using a Quincke spinal needle (BD Spinal needle 20-gauge, 9 cm; Becton Dickinson SA, Madrid, Spain). In dogs receiving PRF, this was performed prior to the foraminal injection, using the same radiofrequency cannula (CC Straight 20-gauge, 10 cm, 10 mm tip; Boston Scientific, Valencia, CA, USA). Motor stimulation was used to optimise cannula positioning, aiming for a positive motor response at ≥ 0.75 V and impedance below 400 *Ω*. PRF was delivered using a Neurotherm NT1100 unit (Croydon, England), set to default parameters: 20 ms pulse width, 2 Hz frequency, 42 °C temperature, and 45 V for a duration of 2 min.

Following the foraminal procedure, an epidural injection was administered via a sacrococcygeal catheter. Catheter placement was performed using a technique adapted from a previous description for the lumbosacral region in dogs (31). A Tuohy needle (Perifix, Filter Set; BBraun, UK) was advanced in a dorsocaudal to ventrocranial direction toward the dorsal sacrococcygeal space, using an ultrasound-guided in-plane parasagittal approach (S9v Sonoscape Guangdong, China). After puncturing the skin and removing the stylet, the needle was advanced until its tip was visualised just ventral to the interarcuate ligament. A syringe containing 0.5 mL of 0.9% NaCl and 0.5 mL of air was connected to the needle. Following negative aspiration of cerebrospinal fluid or blood, 0.3 mL of NaCl was injected without resistance. Real-time US confirmed epidural placement, demonstrated by collapse and recoil of the dural sac in the lumbosacral region. An epidural catheter prefilled with radiological contrast was then threaded through the Tuohy needle and advanced to the target location under fluoroscopic guidance. Fluoroscopy enabled confirmation of catheter tip placement just caudal to the lumbosacral junction and visualisation of contrast spread during injection.

In all injections (both foraminal and epidural) a radiological contrast study (Iohexol; Omnipaque, 300 mg/mL, GE Healthcare, UK) was performed prior to administering therapeutic agents to confirm accurate needle or catheter placement and appropriate spread of injectate around the target neural structures. Aspiration was routinely performed before injection to exclude inadvertent intravascular or intrathecal placement.

All therapeutic injections consisted of a mixture of dexamethasone (Dexamethasone Sodium phosphate 2 mg/mL, Norbrook Laboratories Ltd., Northen Ireland, or Dexamethasone 3.8 mg/mL solution for injection; Aspen Pharma Trading Limited, Ireland) and bupivacaine (Bupivacaine Hydrochloride 0.25–0.5% solution for injection; Mercury Pharmaceuticals Ltd., UK).

### 2.3 Outcome measures

Pain severity was assessed during clinical examination using the LSPain Scale, a behavioural-based scale specifically designed for the assessment of lumbosacral pain in dogs (32). Following an initial neurological examination performed by a board-certified neurologist, all clinical pain scores were performed by the attending board-certified anaesthetist performing the procedures. In parallel, caregivers completed the Canine Brief Pain Inventory (CBPI), a metrology instrument for chronic pain assessment in dogs, currently validated for bone cancer pain and osteoarthritis (33, 34), which contains pain severity scores (PSS), pain interference scores (PIS), and a categorical assessment of QoL.

LSPain Scale scores and QoL were recorded as ordinal categorical variables, while PSS and PIS were treated as continuous variables. Pain severity and QoL were assessed at three time points: baseline, first post-procedure, and second post-procedure assessment. Duration of clinical improvement was recorded in months, with the observation period set at 24 months for dogs that continued to show sustained improvement throughout the follow-up.

Treatment was considered effective (clinically significant) when both of the following criteria were met: a two-grade improvement in LSPain Scale scores and a clinically significant reduction in CBPI scores, defined as a decrease of ≥1 point in PSS and ≥2 points in PIS (35). Pain recurrence was defined as the loss of either criterion. In cases where CBPI scores or clinical re-examination at the institution were unavailable, recurrence was still considered present if it was reported by the caregiver or referring veterinary surgeon.

### 2.4 Statistical analysis

All analyses were conducted using R (version 4.4.1) and RStudio (version 2024.04.2 + 764). Descriptive statistics were used to summarise patient demographics, clinical variables, and outcome distributions.

The analysis aimed to compare baseline clinical pain severity (LSPain Scale scores), CBPI-based scores and QoL between treatment groups (No-PRF and PRF), assess within-group changes in these outcomes over time, and evaluate the trajectory and duration of improvement following interventional pain management. The timing of the first and second clinical assessments was also compared between the No-PRF and PRF groups to ensure that both cohorts were evaluated at equivalent follow-up intervals. Duration of improvement was defined as the time from the procedure until documented pain recurrence or censoring at 24 months in cases without recurrence. Longitudinal changes in Canine Brief Pain Inventory (CBPI) scores were analysed over a 24-week period.

Statistical methods were selected based on the scale, structure, and distribution of each variable. Comparative analysis between and withing groups for categorical variables used the Fisher’s Exact Test, Mann–Whitney U Test and Wilcoxon Signed-Rank as appropriate. Longitudinal CBPI data were analysed using Generalised Linear Mixed Models (GLMMs) to account for repeated measures, irregular follow-up intervals, and within-subject variability. Final model choice was guided by information criteria and diagnostic evaluation of model assumptions. Statistical significance was defined as *p* < 0.05. Model performance was assessed using the Akaike Information Criterion (AIC), Bayesian Information Criterion (BIC), and residual diagnostics. For model-based analyses, 95% confidence intervals were obtained using non-parametric bootstrapping. Results are presented as counts and percentages, median (range), or mean ± standard deviation, as appropriate. For regression models, estimates are reported with standard errors, confidence intervals, and *p*-values.

## 3 Results

### 3.1 Case selection

**Figure 1:**
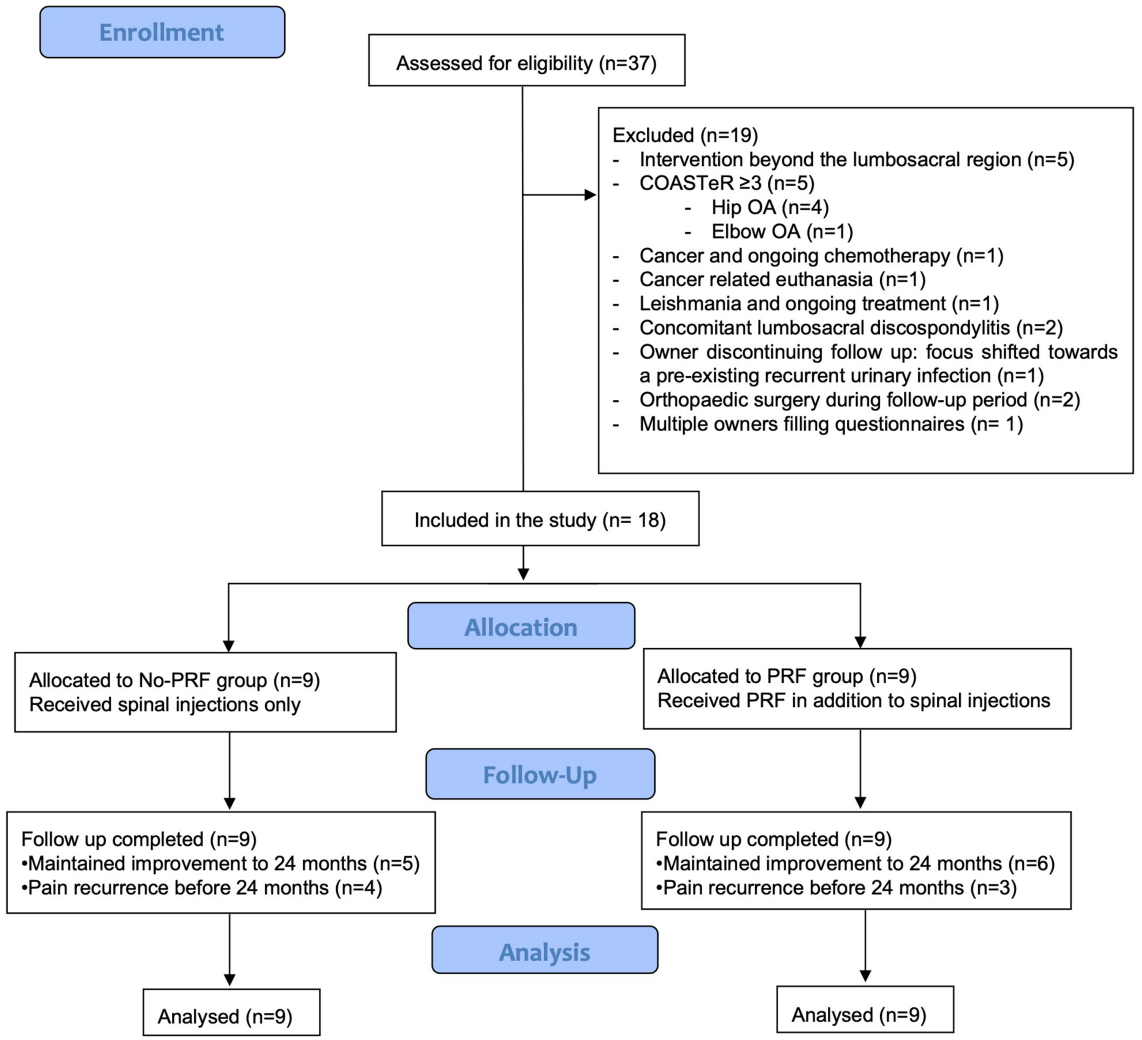
Flow diagram showing eligibility assessment, exclusion criteria, and group allocation for dogs undergoing interventional management of lumbosacral pain. Eighteen dogs met inclusion criteria and received spinal injections with (PRF group) or without (No-PRF group) pulsed radiofrequency. Follow-up completion and inclusion in the final analysis are shown.

A total of 18 dogs met the inclusion–exclusion criteria and were included in the final analysis. A flow diagram depicting case selection, group allocation, follow-up, and analysis is presented in Figure 1. The group No-PRF was composed by two Hungarian Vizsla, two French Bulldog, one Labrador Retriever, one German Shepherd dog, one Pug, one Italian Spinone, and one Schnauzer Miniature. All dogs in the No-PRF had a median of 23.8 kg (9.2–55.2 kg) and a median body condition score of 6/9 (5–7/9) according to the World Small Animal Veterinary Association (WSAVA) Classification (36). The group PRF consisted of two Cocker Spaniel, one Hungarian Vizsla, one French Bulldog, one Golden Retriever, one Giant Schnauzer, one Border Collie and two large-crossbreed dogs. The median weight in this group was 21.1 kg (12–37 kg) and the body condition score 6/9 (6–7/9). Age, pain duration and severity, pre- and post-procedure medications and MRI-based diagnoses are summarised in Table 1.

**Table 1:** Demographics, duration of pain, medication status, diagnosis, procedure, and outcomes in 18 dogs undergoing interventional pain management for lumbosacral pain.

Case	Age at procedure (months)	Pain pre-procedure (months)	Pre-procedure pain severity	Medication (pre-procedure)	MRI diagnosis	Procedure	Medication (end-study)	Improvement post-procedure (months)	Clinically significant improvement post-procedure (months)
1	59	6	Moderate	Gabapentin, meloxicam^a^	L7-S1 spondylosis deformans with left foraminal stenosis and left L7 radiculopathy	Left L7 foraminal (0.1 mg/kg D + 0.05 mL B 0.2%) + SC epidural catheter (0.1 mg/kg D + 0.05 mL B 0.2%) injections	Gabapentin, meloxicam	6.1	6.1
2	26	5.5	Moderate	Gabapentin	L7-S1 IVD extrusion with cauda equina and left foraminal stenosis^c^	Left L7 foraminal (0.1 mg/kg D + 0.05 mL B 0.2%) + SC epidural catheter (0.1 mg/kg D + 0.05 mL B 0.2%) injections	None	21.2	0
3	67	3	Moderate	Pregabalin	L7-S1 IVD extrusion with cauda equina, left foraminal stenosis and left L7 radiculopathy	Left L7 foraminal (0.1 mg/kg D + 0.05 mL B 0.2%) + SC epidural catheter (0.1 mg/kg D + 0.05 mL B 0.2%) injections	None	>24	0
4	71	1.5	Moderate	Gabapentin, paracetamol	L7-S1 IVD protrusion with cauda equina and bilateral foraminal stenosis	Bilateral L7 foraminal (0.1 mg/kg D + 0.05 mL B 0.2%) + SC epidural catheter (0.1 mg/kg D + 0.05 mL B 0.2%) injections	None	16.5	16.5
5^b^	112	36	Mild	Gabapentin, paracetamol, galliprant	L7-S1 IVD extrusion with cauda equina, bilateral foraminal stenosis and bilateral L7 radiculopathy	Bilateral L7 foraminal (0.1 mg/kg D + 0.05 mL B 0.2%) + SC epidural catheter (0.1 mg/kg D + 0.05 mL B 0.2%) injections	Gabapentin, bedinvetmab	1.2	0
6	37	2	Severe	Gabapentin, meloxicam^a^, paracetamol	L7-S1 IVD protrusion with bilateral foraminal stenosis and right L7 radiculopathy	Bilateral L7 foraminal (0.1 mg/kg D + 0.05 mL B 0.2%) + SC epidural catheter (0.1 mg/kg D + 0.05 mL B 0.2%) injections	Gabapentin	20.5	20.5
7	130	72	Severe	Gabapentin, robenacoxib^a^, tramadol	L7-S1 spondylosis and IVD protrusion with bilateral foraminal stenosis and bilateral L7 radiculopathy	Bilateral L7 foraminal (0.1 mg/kg D + 0.05 mL B 0.2%) + SC epidural catheter (0.1 mg/kg D + 0.05 mL B 0.2%) injections	Gabapentin, robenacoxib^e^, tramadol	0	0
8	41	4	Moderate	Gabapentin, prednisolone^a^	L7-S1 spondylosis deformans with bilateral foraminal stenosis and bilateral L7 radiculopathy	Bilateral L7 foraminal (0.1 mg/kg D + 0.05 mL B 0.2%) + SC epidural catheter (0.1 mg/kg D + 0.05 mL B 0.2%) injections	None	13.3	13.3
9	107	9	Severe	Gabapentin, meloxicam^a^	L7-S1 IVD protrusion with cauda equina	Bilateral^d^ L7 foraminal (0.1 mg/kg D + 0.05 mL B 0.2%) + SC epidural catheter (0.1 mg/kg D + 0.05 mL B 0.2%) injections	None	>24	>24
10	25	24	Severe	Gabapentin, paracetamol, fluoxetine	L7-S1 IVD degeneration with dynamic cauda equina and bilateral foraminal stenosis	Bilateral L7 foraminal (0.1 mg/kg D + 0.05 mL B 0.2%) + SC epidural catheter (0.1 mg/kg D + 0.05 mL B 0.2%) injection	Gabapentin, fluoxetine	>24	>24
11	96	8	Moderate	Gabapentin	L7-S1 IVD protrusion with cauda equina and right foraminal stenosis	Bilateral^d^ L7 PRF + Bilateral L7 foraminal (0.1 mg/kg D + 0.05 mL B 0.15%) + SC epidural catheter (0.1 mg/kg D + 0.05 mL L 0.15%) injections	None	23.6	23.6
12	42	19	Severe	Gabapentin, meloxicam	L7-S1 IVD degeneration with protrusion, cauda equina, right foraminal stenosis and right L7 radiculopathy	Bilateral^d^ L7 PRF + Bilateral L7 foraminal (0.1 mg/kg D + 0.05 mL B 0.15%) + SC epidural catheter (0.1 mg/kg D + 0.05 mL B 0.15%) injections	None	>24	23.4
13	52	7	Severe	Gabapentin, robenacoxib^a^, paracetamol	L7-S1 spondylosis deformans with right foraminal stenosis and right L7 radiculopathy	Bilateral^d^ L7 PRF + Bilateral L7 foraminal (0.1 mg/kg D + 0.05 mL B 0.15%) + SC epidural catheter (0.1 mg/kg D + 0.05 mL B 0.15%) injections	None	>24	>24
14	66	7	Severe	Meloxicam	L7-S1 spondylosis deformans and protrusion with bilateral foraminal stenosis and bilateral L7 radiculopathy	Bilateral L7 PRF + Bilateral L7-S1 foraminal (0.1 mg/kg D + 0.05 mL B 0.15%) + SC epidural catheter (0.1 mg/kg D + 0.05 mL B 0.15%) injections	None	12.9	2.2
15	134	16	Severe	Pregabalin, enflicoxib^a^, bedinvetmab, paracetamol	L7-S1 spondylosis deformans and IVD protrusion with cauda equina and left foraminal stenosis	Left L7 PRF + Left L7 foraminal (0.1 mg/kg D + 0.05 mL B 0.15%) + SC epidural catheter (0.1 mg/kg D + 0.05 mL B 0.15%) injections	Pregabalin, enflicoxib, bedinvetmab, paracetamol	9.3	9.3
16	126	7	Severe	Gabapentin, paracetamol	L7-S1 spondylosis deformans and IVD extrusion with cauda equina, bilateral foraminal stenosis and bilateral L7 radiculopathy	Bilateral L7 PRF + Bilateral L7 foraminal (0.1 mg/kg D + 0.05 mL B 0.15%) + SC epidural catheter (0.1 mg/kg D + 0.05 mL B 0.15%) injections	Gabapentin	12.8	12.8
17	77	24	Moderate	Gabapentin, paracetamol	L7-S1 degeneration with IVD protrusion, cauda equina, left foraminal stenosis and left L7 radiculopathy	Left L7 PRF + Left L7 foraminal (0.1 mg/kg D + 0.05 mL B 0.15%) + SC epidural catheter (0.1 mg/kg D + 0.05 mL B 0.15%) injections	None	>24	>24
18	48	3	Moderate	Gabapentin	L7-S1 degeneration with IVD protrusion, cauda equina, right foraminal stenosis and right L7 radiculopathy	Bilateral L7 PRF + Bilateral L7 foraminal (0.1 mg/kg D + 0.05 mL B 0.15%) + SC epidural catheter (0.1 mg/kg D + 0.05 mL B 0.15%) injections	Gabapentin	4.4	4.4

a this drug was stopped prior to the procedure.

b patient underwent a bilateral L7-S1 foraminotomy 11 months prior to the procedure.

c imaging diagnosis established by CT instead of MRI.

d treatment was administered bilaterally in accordance with clinical examination.

e this drug was restarted 6 days following the procedure.

B, bupivacaine; D, dexamethasone; IVD, intervertebral disc; L, lidocaine; L7, seventh lumbar vertebra; MRI, magnetic resonance imaging study; PRF, pulsed radiofrequency treatment.

### 3.2 Procedures

All procedures were performed under a non-standardised general anaesthesia protocol tailored to each patient. Premedication agents included methadone (18/18; 100%), medetomidine (14/18; 77.8%) and dexmedetomidine (3/18; 16.7%). All dogs were preoxygenated prior to induction of anaesthesia, which was achieved with intravenous propofol to effect, followed by tracheal intubation with a cuffed endotracheal tube. Anaesthesia was maintained with isoflurane in a mixture of oxygen and air. Mechanical ventilation was initiated if clinically indicated during the procedure. All dogs received intravenous fluid therapy with Ringer’s Lactate during anaesthesia and was resumed upon eating once the dog was bright during the recovery phase. Paracetamol was administered intravenously either during the procedure or during the recovery phase. Dexamethasone doses ranged from 0.2 to 0.3 mg/kg, and bupivacaine concentration used ranged from 0.15 to 0.2%. Table 1 provides a summary of procedural details and duration of improvement for the 18 dogs included in the study.

Transforaminal contrast uptake was achieved in 22 of 28 foraminal injections (78.6%), with all 28 injections (100%) resulting in paravertebral foraminal uptake. Vascular uptake during the initial contrast study was observed in 3 of 28 foraminal injections (10.7%). Subarachnoid uptake was identified in 4 of 28 injections (14.3%). In all cases where intravascular or subarachnoid uptake was detected, the cannula was repositioned before administering the therapeutic agents. No adverse clinical effects were observed in the patients where intravascular or subarachnoid uptake occurred. Although partial diffusion of the bupivacaine–dexamethasone combination into these compartments could not be definitively excluded, clinical outcomes remained favourable. Epidural catheter injections resulted in epidural uptake in all procedures 18/18 (100%), with vascular uptake found in 1/18 procedures (5.6%). In all cases where intravascular uptake was detected, the catheter was repositioned before administering the therapeutic agents.

All but two dogs were discharged on the same day as the procedure. In one case, the dog remained hospitalised overnight due to a late procedure finish. In the other, the dog was kept for close monitoring after developing ipsilateral pelvic limb motor deficits (Dog 4). The patient was discharged the following day, and the caregiver reported progressive resolution of the deficit over 3 days. This event was attributed to a suspected partial intraneural injection. Transient increased urination the night of the procedure was reported in 2/18 patients (11.1%).

### 3.3 Clinical pain assessment (LSPain scale)

Fisher’s Exact Test was used to compare baseline pain severity between the PRF and No-PRF groups. No statistically significant difference was detected (*p* = 0.6372), indicating comparable initial pain levels. From baseline to the first assessment, the PRF group had a median interval of 4 weeks (4–6), while the No-PRF group had a median of 5 weeks (4–6), with no significant difference between groups (*p* = 0.6667). From the first to the second assessment, the PRF group had a median of 5 weeks (4–7) and the No-PRF group had 6 weeks (4–6), again with no significant difference (*p* = 0.474). Within-group changes over time were assessed using the Wilcoxon Signed-Rank Test. In the No-PRF group, pain severity significantly decreased from baseline to the first assessment (*p* = 0.0061) and remained significantly improved at the second assessment (*p* = 0.0177), with no further change between the first and second assessments (*p* = 1.000). Similarly, the PRF group showed a significant reduction in pain severity from baseline to the first assessment (*p* = 0.0069) and from baseline to the second assessment (*p* = 0.0074), with no significant change between the first and second assessments (*p* = 0.4237). These results suggest that the most substantial clinical improvement occurred early after the procedure and was maintained over time in both groups.

To compare the magnitude of improvement between treatment groups, the change in pain severity from baseline to the second assessment was analysed using the Mann–Whitney U test. Although the PRF group showed a numerically greater reduction in pain severity, with a median improvement of 3 points (range: 2–3) compared to 2 points (range: 2–3) in the No-PRF group, this difference did not reach statistical significance (W = 23, *p* = 0.3253).

**Figure 2:**
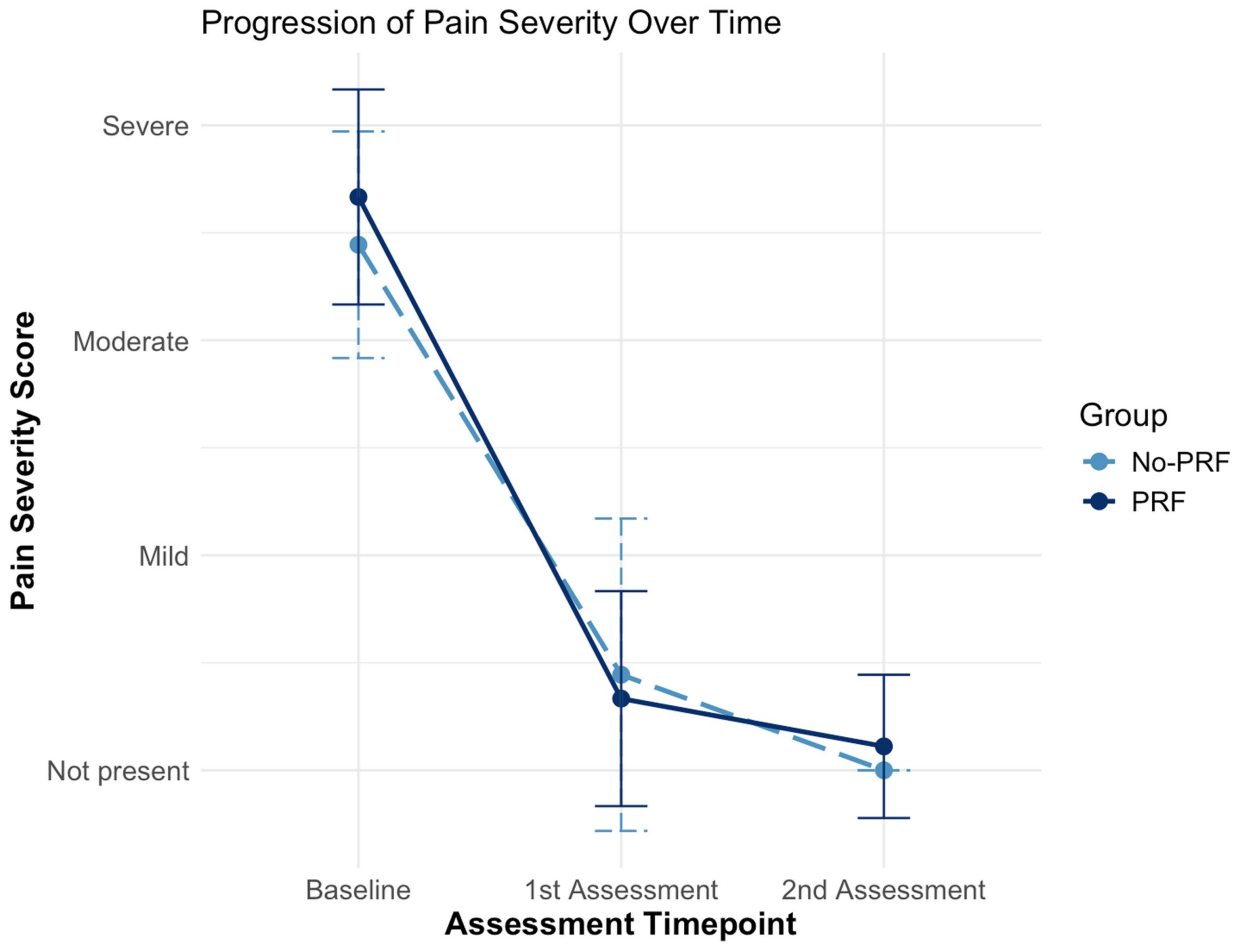
Boxplot illustrating changes in clinical pain severity scores (LSPain Scale) at baseline, first follow-up, and second follow-up in dogs treated with image-guided spinal injections, with or without pulsed radiofrequency (PRF). Both groups showed a statistically significant reduction in pain severity from baseline to first and second follow-up (*p* < 0.01), with no further change thereafter. The PRF group demonstrated a numerically greater improvement, although the between-group difference was not statistically significant.

The progression of mean pain severity scores over time is depicted in Figure 2, which illustrates a rapid decline in pain scores immediately following treatment in both groups, with minimal changes thereafter.

### 3.4 Quality of life (CBPI-based)

Fisher’s Exact Test was used to compare baseline QoL scores between the PRF and No-PRF groups. Although a greater proportion of dogs in the PRF group presented with poorer QoL, this difference did not reach statistical significance (*p* = 0.3348), indicating that both groups were comparable at baseline. Changes over time within each group were analysed using the Wilcoxon Signed-Rank Test. In the No-PRF group, QoL significantly improved from baseline to the first assessment (*p* = 0.0107) and remained improved at the second assessment (*p* = 0.0107), with no additional change between post-treatment time points (no score changes observed; *p* not computed). In the PRF group, QoL also improved significantly from baseline to the first (*p* = 0.0206) and second assessments (*p* = 0.0103), with no further change between follow-ups (*p* = 0.5862). These results indicate that the greatest QoL improvement occurred early and was maintained over time in both treatment groups.

Dogs treated with PRF experienced a significantly greater overall improvement in QoL scores compared to those receiving injections alone. A Mann–Whitney U test demonstrated that this difference was statistically significant (*p* = 0.0247), with a median improvement of 2 points (range: 0–2) in the PRF group versus 1 point (range: 0–1) in the No-PRF group. One dog in each group did not experience any change in QoL following treatment (dogs 7 and 18).

**Figure 3:**
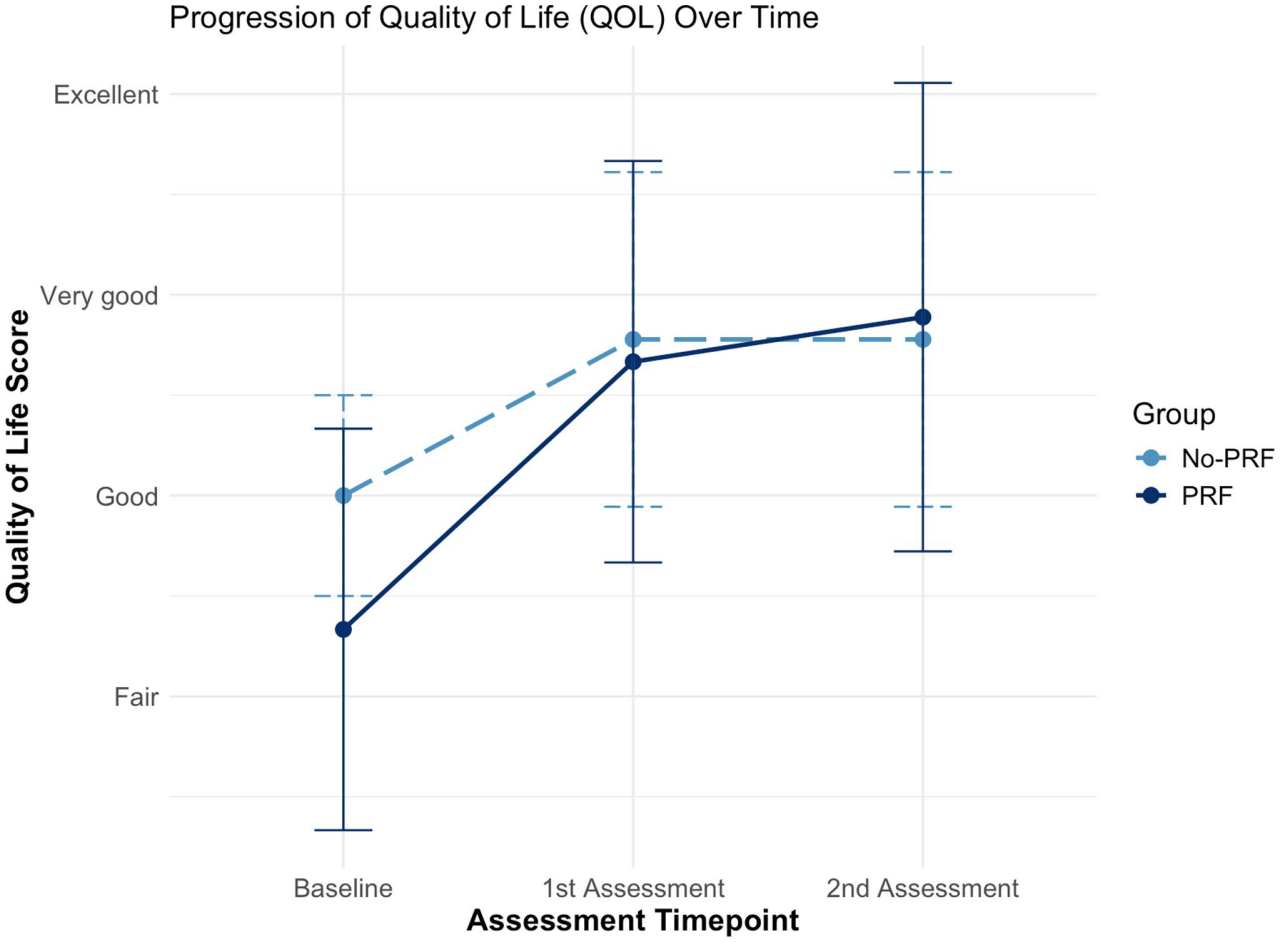
Boxplot depicting changes in quality of life (QoL) scores at baseline, first follow-up, and second follow-up in dogs treated with image-guided spinal injections, with or without pulsed radiofrequency (PRF). Both groups showed a statistically significant improvement from baseline to follow-up (*p* < 0.05), sustained over time. The PRF group exhibited a significantly greater overall improvement compared to the No-PRF group (*p* = 0.0247).

The evolution of mean QoL scores over time is illustrated in Figure 3, which shows a marked early improvement following treatment in both groups, with minimal change thereafter.

### 3.5 CBPI scores

CBPI scores were analysed using a GLMM to assess longitudinal trends in pain reduction following treatment. The model included fixed effects for time (Week), treatment group (PRF vs. No-PRF), and their interaction, along with a random intercept for individual patients.

Over the 24-week follow-up period, CBPI scores showed a consistent and progressive decline in both treatment groups (*β* = −1.68, SE = 0.45, *p* = 0.0005). No significant differences were observed between the PRF and No-PRF groups at baseline (β = 8.29, SE = 10.22, *p* = 0.426) or during the study period (β = −0.21, SE = 0.57, *p* = 0.721), suggesting that the rate of improvement over time was similar in both groups.

Model diagnostics supported the robustness of the GLMM approach. Residuals were approximately normally distributed (Shapiro–Wilk *p* = 0.1175), with no evidence of heteroscedasticity. The random intercept variance (325.0; 95% CI: 9.26 to 25.17) confirmed substantial between-patient variability. Furthermore, the GLMM outperformed the fixed-effects model in terms of information criteria (AIC: 538.43 vs. 566.53; BIC: 551.19 vs. 577.17), validating the appropriateness of the mixed-effects structure.

**Figure 4:**
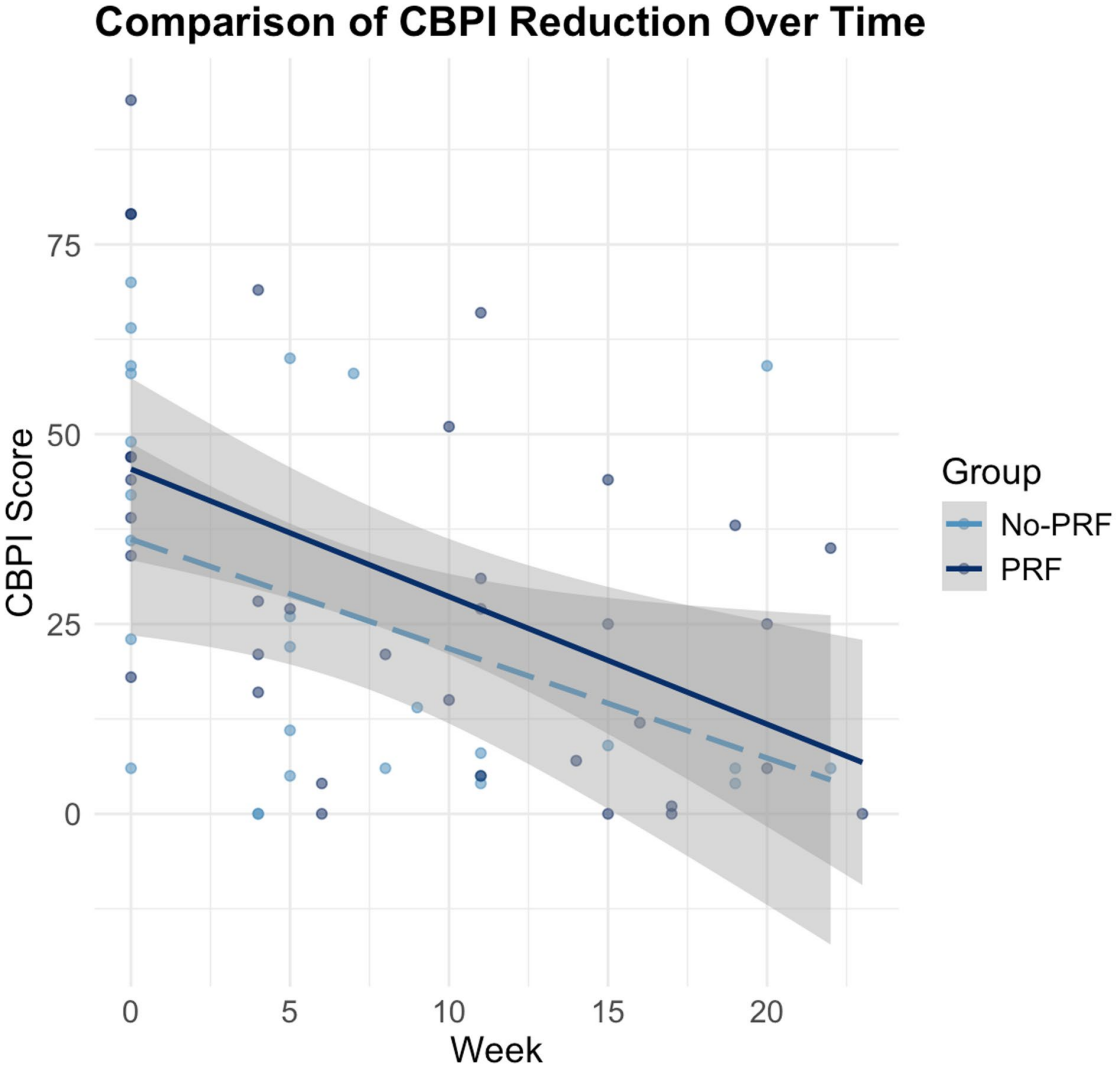
Scatter plot with fitted regression lines showing the reduction in Canine Brief Pain Inventory (CBPI) scores over 24 weeks in dogs treated with image-guided spinal injections, with or without pulsed radiofrequency (PRF). Lines represent estimated marginal means from a generalized linear mixed model with 95% confidence intervals. Both groups exhibited a statistically significant decline in CBPI scores over time (*p* = 0.0005). The PRF group demonstrated a steeper rate of improvement, although the difference in slope between groups did not reach statistical significance.

Although both treatment groups demonstrated a statistically significant reduction in CBPI scores, no significant difference in the magnitude or rate of improvement was observed between groups. The evolution of CBPI scores is illustrated in Figure 4, which shows the linear decline in pain scores for each group with overlapping confidence intervals, reflecting parallel trajectories of improvement.

### 3.6 Proportion of clinically significant responders

Based on the LSPain Scale alone, 9/9 dogs (100%) in the PRF group and 8/9 dogs (88.9%) in the No-PRF group met criteria for clinically significant improvement (dog 7 did not meet the threshold). Regarding CBPI scores, improvement was observed in 9/9 dogs (100%) in the PRF group and 8/9 dogs (88.9%) in the No-PRF group (dog 7 not showing an improvement). However, only in 5/9 dogs (55.5%) in the No-PRF group (dogs 2, 3 and 5 not experiencing a clinically significant improvement) met the predefined criteria. As a result, 9/9 dogs (100%) in the PRF group qualified as responders, compared to 5/9 dogs (55.5%) in the No-PRF group.

Fisher’s Exact Test yielded a *p*-value of 0.082, indicating that the between-group difference in responder rates did not reach statistical significance. The odds ratio was estimated as infinite (95% CI: 0.79 to ∞), reflecting the absence of non-responders in the PRF group.

### 3.7 Duration of improvement

**Figure 5:**
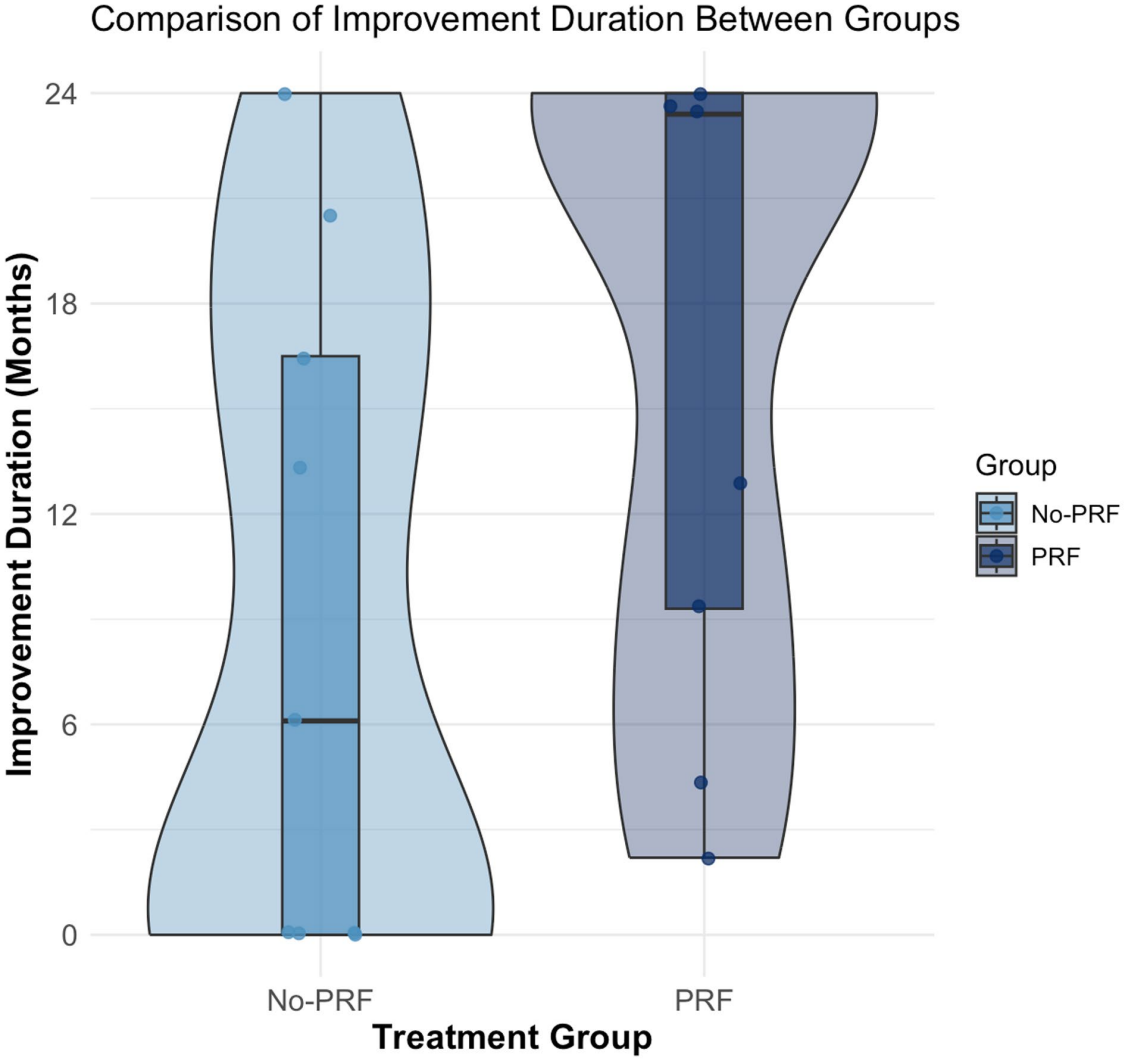
Violin plot with kernel density estimation (KDE) illustrating the distribution of clinically significant improvement duration (months) in dogs treated with image-guided spinal injections, either with pulsed radiofrequency (PRF group) or without (No-PRF group). The KDE is truncated at 24 months, the study’s maximum follow-up. Overlaid boxplots show median and interquartile ranges for each group, with individual data points displayed. Although the PRF group visually trends toward longer durations, this difference did not reach statistical significance.

The Shapiro–Wilk test confirmed that duration of improvement followed a non-normal distribution in both subgroups (general improvement: W = 0.848, *p* = 0.0079; clinically significant improvement: W = 0.839, *p* = 0.0058). Comparisons between the PRF and No-PRF groups were performed using the Mann–Whitney U test. Although the PRF group showed a greater duration of the improvement, with a median duration was 17.7 months (range: 4.4 to 24) compared to 14.1 months (range: 0 to 24) in the No-PRF group, this difference was not statistically significant (W = 24.5, *p* = 0.1610; estimated difference in location: 4.8 months; 95% CI: −17.9 to 3.5). For clinically significant improvement, the PRF also showed a greater duration of 16.4 months (range: 2.2 to 24) versus 8.9 months (range: 0 to 24) in the No-PRF. Again, the difference between groups did not reach statistical significance (W = 21.5, *p* = 0.0988; estimated difference in location: 7.5 months; 95% CI: −23.4 to 0.6). A graphical representation of clinically significant improvement duration is shown in Figure 5, illustrating the distribution of outcomes between treatment groups. No transient, self-limiting pain episodes following a traumatic event were reported in any dog during the study period.

## 4 Discussion

To our knowledge, this is the first clinical report describing the use of foraminal injections in dogs with naturally occurring lumbosacral stenosis, extending previous anatomical and experimental work in cadavers and healthy animals. It is also the first to report the clinical application of PRF at the DRG as a neuromodulation modality in veterinary medicine.

This study evaluated the long-term outcomes of image-guided interventional pain management in dogs with lumbosacral pain unresponsive to rest and oral medication. All dogs were treated using a combination of fluoroscopy- and ultrasound-guided foraminal and epidural injections, with or without the addition of PRF targeting the DRG. These interventions resulted in clinically significant and sustained pain relief in most cases, supporting the therapeutic value of image-guided interventional techniques for managing lumbosacral pain in dogs.

Across all outcome measures (LSPain Scale scores, QoL, CBPI scores, proportion of responders, and duration of improvement), the PRF group demonstrated more favourable outcomes. These results are consistent with human data supporting the additive effect of PRF in similar clinical contexts (25–29). However, among these measures, only QoL reached statistical significance.

Given PRF’s favourable safety profile and its documented clinical benefit in humans, it would be unwise to dismiss its potential solely based on the absence of statistically significant differences in this small cohort. At a minimum, the results suggest that both treatment approaches were similarly effective over the long term. However, the consistently more favourable trends observed in the PRF group across all outcome measures raise the possibility of a type II error, defined as the failure to detect a true difference when one genuinely exists (37). These findings support further investigation of PRF as an adjunctive modality in prospective studies aimed at optimising pain management in dogs with naturally occurring lumbosacral pain.

At the time of study design, no published clinical data were available on the use of image-guided foraminal injections, with or without PRF, in dogs with naturally occurring lumbosacral pain. Therefore, *a priori* sample size calculation and power estimation was not possible. Post-hoc power analysis was deliberately not performed due to its conceptual and statistical limitations. Specifically, it is considered unreliable because it is mathematically redundant with the *p*-value, provides no reliable insight into the probability of a type II error, and relies on observed effect sizes that are subject to high variability in small samples (38). Instead, the results of this study should be used to inform sample size estimations and study design for future prospective studies involving similar populations and using comparable outcome measures. To contextualise the current results, if the current LSPain Scale scores were used to estimate the sample size required to detect the observed treatment effect in future studies, a total of 22 dogs (11 per group) would be needed to achieve 80% power at a 5% significance level, based on the Mann–Whitney U test and an observed Cliff’s Delta of 0.556. Given the smaller number of animals included in this study, the absence of statistical significance is not unexpected. In this study, the PRF group demonstrated a median improvement of one grade greater than the No-PRF group. Although this difference did not reach statistical significance, such a shift may represent a clinically meaningful change, especially in a four-point ordinal scale where a one-grade improvement reflects a perceptible gain in comfort.

Because CBPI scores were collected at irregular time points over the 24-week period, GLMMs were used to model pain trends while accounting for repeated measures and individual variability. Although the raw CBPI scores were not normally distributed, the use of GLMMs was justified by the normal distribution of residuals (Shapiro–Wilk *p* = 0.1175) and the absence of heteroscedasticity, as model assumptions are evaluated on residuals rather than the original data (39). The inclusion of random intercepts improved model fit, as indicated by lower AIC and BIC values compared to fixed-effects models, demonstrating a better balance between explanatory power and model complexity (40).

While in this case GLMMs were well suited for the irregular follow-up, future studies should consider paired comparisons of CBPI scores collected at fixed, standardised timepoints. This prospective approach would reduce within-subject variability, enhance consistency in longitudinal assessments, and increase statistical power to detect true differences between treatment groups (41). The findings of this study provide a foundation for future trial design when considering PRF as an additive intervention. Importantly, when evaluating a safe adjunctive modality such as PRF, even modest clinical benefits may be sufficient to support its inclusion in multimodal treatment protocols.

Lumbosacral interlaminar epidural corticosteroid injections have demonstrated favourable short- to mid-term outcomes in cohorts of dogs with lumbosacral pain unresponsive to pharmacological treatment (3, 8). However, direct comparison of results across studies remains challenging due to methodological variability, including differences in study design, treatment protocols, outcome definitions, follow-up duration, and assessment strategies. None of the available studies, including ours, employed blinded outcome assessment. In retrospective studies, this is inherently challenging, as the clinician performing the intervention is typically responsible for both treatment and follow-up. Although prospective designs offer opportunities for blinded assessment, this is not always incorporated in clinical research. As a result, the potential for caregiver and clinician placebo effects must be acknowledged (42). Nevertheless, this limitation does not necessarily compromise the clinical relevance of the outcomes reported, which reflect real-world settings and decision-making processes.

The results of the present study compare favourably with these earlier reports, in which pain recurrence following lumbosacral interlaminar corticosteroid injections was common, often occurring within days to months (3, 8). Janssens et al. (3) reported the longest documented follow-up to date in dogs treated with epidural corticosteroids for lumbosacral pain (ranging from 5 to 66 months). Their study included 38 dogs, diagnosed with lumbosacral stenosis, that had failed conservative management with rest and oral medication. Despite receiving repeated epidural corticosteroid injections, relapse was frequent. Self-limiting polyuria, polydipsia, and worsening of pain were identified in 19/38 (50%) dogs following the epidural injection. Transient polyuria was attributed to systemic corticosteroid absorption. In our cohort, transient increased urination was most likely associated with dexamethasone administration, although the use of alpha two agonists and peri anaesthetic fluid therapy may also have contributed.

The median duration of effect following the first injection was 11 days, which increased to 4.5 months after the third injection. By the end of the follow-up period, 79% of caregivers reported some degree of pain improvement, and 53% reported complete resolution of clinical signs. However, clinical assessments were not standardised, and outcomes were based exclusively on caregiver feedback. This was largely due to the involvement of multiple assessors and the absence of a structured methodology for clinical evaluation. In a prospective study, Gomes et al. (8) evaluated 32 dogs with MRI-confirmed degenerative lumbosacral stenosis, all of which had failed prior conservative treatment. Each dog received a single lumbosacral epidural corticosteroid injection under general anaesthesia, with neurostimulation used to confirm needle placement. Initial clinical improvement was observed in 27/32 of dogs (84.4%). However, 77.2% of patients where follow up was available (17/22) experienced relapse within a median of 2 months (range: 0.5 to 6 months). Sustained improvement without recurrence was documented in only 5 out of 32 (15.6%) over a mean follow-up period of 9.4 months. No major complications were reported. (3)Outcomes were assessed by a board-certified neurologist during follow-up consultations using a structured, categorical clinical outcome assessment based on non-validated criteria. As in the study by Janssens et al. (3), caregiver feedback was obtained using non-validated questionnaires.

These studies highlight the lack of standardised, validated outcome measures for assessing lumbosacral pain in dogs. The need for formal validation of pain assessment tools tailored to specific pain conditions in dogs has been consistently emphasised by expert panels, as reflected in the 2017 (43) and 2025 (44) Pain in Animals Workshop (PAW) reports. The LSPain Scale represents a step towards addressing this gap as the first behaviour-based, clinician-administered instrument specifically designed to assess lumbosacral pain in dogs. It uses a four-point ordinal scale to grade behavioural responses to palpation and has undergone initial validation, demonstrating excellent convergent and discriminant validity, responsiveness to treatment, inter and intra rater reliability, and usability across varying levels of clinical experience (32).

The 2025 PAW report also underscored the importance of selecting outcome measures that are aligned with the specific study goals, recognising that different tools assess distinct domains. This supports the complementary use of clinician-based and caregiver-reported instruments, such as the LSPain Scale and the CBPI, when evaluating pain and its impact on QoL, in contrast to measures primarily focused on lameness or biomechanical function. In the present study, the LSPain Scale provided a standardised measure of clinician-assessed pain severity and treatment response, complementing the caregiver-reported CBPI to enhance methodological robustness through the integration of both clinician and caregiver perspectives.

A distinctive feature of this study was the application of a stringent, dual-criterion definition for treatment success, requiring both a two-grade improvement in clinical pain score and a clinically meaningful reduction in CBPI scores. Pain recurrence was recorded when either criterion was no longer met or when deterioration was reported by caregivers or referring veterinarians. This approach aimed to minimise false positives in identifying treatment responders, while allowing early detection of clinical relapse. It ensured that genuine responders were reliably identified and even subtle deteriorations prompted clinical attention. The impact of this methodology is reflected in the outcomes: whilst 100% of dogs in the PRF group and 88.9% of dogs in the no-PRF group showed improvement in both clinical and CBPI scores, only 55.5% of dogs in the No-PRF group met the full criteria for clinically relevant improvement, compared to 100% in the PRF group.

Several methodological aspects, beyond the neuromodulatory effects of the PRF intervention itself, may have contributed to the favourable outcomes observed. One important factor lies in the approach to peri-procedural medication. In this study, oral analgesics were continued following the intervention, consistent with the approach described by Gomes et al. (8). This strategy aimed to reduce confounding in the assessment of treatment response, as abrupt medication withdrawal could result in rebound pain and misrepresent the actual efficacy of the procedure. Additionally, interventional procedures were integrated as part of a broader multimodal analgesic plan, serving as a supportive “handrail” within the analgesic ladder (45). Medications were tapered cautiously and only once pain levels had clearly diminished, particularly in dogs with chronic refractory pain and suspected central sensitisation.

Although Gomes et al. (8) also maintained oral analgesics following the procedure, the specific drugs used were not reported in detail and were described only as “non-standardised.” Despite reporting a greater short-term efficacy compared to Janssens et al. (3), the long-term resolution rate was lower. Only 15.6% of dogs in the Gomes et al. (8) cohort achieved sustained improvement after a single injection, whereas 53% of caregivers in the Janssens et al. (3) study reported complete resolution of clinical signs by the end of the follow-up period. It is important to note that the Janssens protocol involved repeated epidural corticosteroid injections, in contrast to the single injection approach used in the Gomes’ study. This difference may reflect a cumulative or synergistic effect of repeated epidural corticosteroid administration in achieving durable pain relief.

In our study, baseline medication use was not standardised due to the retrospective design. The variability in pharmacological treatment reflects real-world clinical practice but may also contribute to variability in treatment response. This should be controlled where possible in future prospective studies to more clearly determine the independent contribution of each intervention. At the time of intervention, all dogs were receiving oral analgesics, most commonly gabapentinoids (94.4%), non-steroidal anti-inflammatory drugs (NSAIDs) (44.4%), and paracetamol (44.4%). Less frequently used drugs included grapiprant, bedinvetmab, prednisolone, fluoxetine, and tramadol (each administered in 5.5% of cases). By the end of follow-up, 50% of dogs in both treatment groups remained on oral medication, most commonly gabapentinoids (44.4%) and NSAIDs (16.7%). NSAIDs were discontinued 3–7 days prior to the procedure at clinician’s discretion to reduce gastrointestinal risk associated with concurrent corticosteroid use, and were reintroduced at a later stage if deemed clinically appropriate. This medication profile reflects drugs commonly used in dogs medically treated for lumbosacral stenosis (46). However, none of the dogs in our cohort achieved sufficient pain relief with these therapies alone, suggesting a population with more complex pain mechanisms potentially involving central sensitisation. In such cases, conventional pharmacological strategies may be insufficient, reinforcing the need for targeted, image-guided interventional therapies. This approach likely minimised confounding in outcome assessment and may have helped preserve the analgesic benefit induced by the procedure, contributing to the favourable long-term outcomes observed in our cohort.

Another factor that may have contributed to the observed outcomes was the use of a corticosteroid–local anaesthetic combination. This practice is well established in medicine for epidural interventions targeting the lumbar spine (47). A systematic review and meta-analysis concluded that bupivacaine, whether used alone or in combination with corticosteroids, was effective in alleviating chronic low back pain (47). A subsequent Cochrane-based meta-analysis focusing specifically on lumbar radiculopathy and sciatica reinforced these findings, reporting strong evidence supporting the efficacy of the corticosteroid–local anaesthetic combination and moderate to strong evidence for local anaesthetics alone (48).

An important consideration in foraminal injections, is the choice of corticosteroid. In this study, all dogs received dexamethasone, a non-particulate corticosteroid widely used in human interventional practice for its favourable safety profile. This practice aligns with the American Society of Interventional Pain Physicians (ASIPP) guidelines, which recommend non-particulate agents for transforaminal procedures due to the risk of embolic complications associated with particulate steroids (49). A comprehensive review of complications associated with lumbar transforaminal procedures linked spinal cord infarction secondary to embolic events exclusively to particulate corticosteroids such as methylprednisolone, betamethasone, or triamcinolone (50). In contrast, only two isolated cases of neurological complications have been reported with dexamethasone, neither involving embolism. In neither case was vascular uptake detected on contrast studies, and the proposed mechanisms of injury were unrelated to embolisation. Proposed mechanisms included conus medullaris infarction secondary to vasospasm or vessel dissection (51), or a presumed intra-arterial injection into a radiculomedullary artery, leading to temporary paraplegia, with no lasting neurological sequelae attributable to dexamethasone (52). A recent retrospective study found no adverse events with either steroid type (53) but noted that patients receiving dexamethasone required repeat injections less frequently, with 87.5% maintaining clinical benefit without the need for a second injection over 12 months. Based on these findings, the authors recommended the preferential use of non-particulate corticosteroids, in line with broader evidence linking particulate formulations to embolic complications (50). Although direct veterinary data are currently lacking, these findings support the use of dexamethasone in dogs undergoing transforaminal epidural injections, not only due to its favourable safety profile but also its potential for sustained clinical benefit.

To our knowledge, this is the first report that describes the combined use of bupivacaine and dexamethasone epidurally in dogs with lumbosacral stenosis and associated lumbosacral pain. A similar combination has, however, been recently reported in a cat with lumbosacral disease (54). In the first 10 dogs, bupivacaine 0.2% was selected based on clinical experience, aiming to extend analgesia into the recovery period and allow assessment of lumbosacral pain once the patient was fully alert. A favourable response at this stage, defined as a clear reduction in pain compared with pre-procedure, was considered a useful prognostic indicator, suggesting effective targeting of pain-generating neural structures. Although transient pelvic limb motor deficits were anticipated at this concentration, these were deemed as acceptable given the diagnostic and therapeutic benefits. However, clinical observations in dogs outside the study cohort raised concerns about the potential for longer-lasting motor deficits at this concentration. In response, the bupivacaine concentration was reduced to 0.15% for the remaining eight dogs, with the aim of minimising motor effects while preserving adequate analgesia. This adjustment aimed to improve early detection of motor deficits that might indicate procedural complications such as neurapraxia or partial intraneural injection. One dog receiving the higher concentration developed a transient unilateral pelvic limb motor deficit, suspected to result from partial intraneural injection. While the clinical course was self-limiting and without long-term sequelae, this case reinforces the importance of minimising confounding motor effects to enable more accurate post-procedural monitoring and early identification of complications.

The delivery technique itself may also have contributed to improved outcomes. In medicine, transforaminal epidural injections are preferred over interlaminar approaches for treating radicular pain secondary to lumbosacral disc herniation. This is due to their ability to deliver medication directly into the ventral epidural space at the site of nerve root compression. In contrast, interlaminar injections deposit drugs into the dorsal epidural compartment, relying on passive diffusion to reach the target, which may limit therapeutic efficacy. Meta-analytic data confirm the superior performance of the transforaminal technique in both short- and long-term outcomes, including pain relief and functional improvement (9). Given that lumbosacral disc herniation and radiculopathy have recently been identified as key predictors of lumbosacral pain in dogs (5), it is reasonable to hypothesise that the targeted benefits of the transforaminal technique may translate into improved outcomes in the canine population as well.

Although transforaminal epidural injections have been described in dog cadavers and healthy dogs (16–18), their clinical efficacy in dogs with naturally occurring lumbosacral pain had not previously been evaluated. The foraminal technique employed in this study was based on a cadaveric model (19), where contrast consistently demonstrated accurate distribution to the target spinal nerve and DRG. That study also documented vascular and subarachnoid uptake, highlighting the anatomical complexity of the region and the importance of contrast verification prior to therapeutic injection. In. this clinical cohort, rates of transforaminal epidural and intravascular uptake (78.7 and 10.7%, respectively) closely matched previous cadaveric findings (80% and 10–20%, respectively).

Notably, Liotta et al. reported a modest reduction in subarachnoid spread when performing transforaminal epidurals in live dogs compared to cadavers, decreasing from 25 to 16.6% (16, 17). Similarly, in our study, the incidence of subarachnoid uptake was lower (14.3%) than the 50–60% reported in the cadaveric model, suggesting that patient-specific anatomical or procedural factors may reduce the risk of subarachnoid migration *in vivo*. Several factors may contribute to this discrepancy. First, the absence of cerebrospinal circulation in cadavers may allow contrast medium to accumulate locally, leading to more apparent subarachnoid visualisation. In live animals, cerebrospinal flow may disperse small volumes of contrast, potentially resulting in underestimation of subtle subarachnoid spread. Second, post-mortem changes in tissue integrity could influence the permeability or mechanical resilience of dural membranes, potentially predisposing cadaveric specimens to higher rates of inadvertent subarachnoid uptake. Additionally, greater operator experience and increased familiarity with the technique during the clinical phase may have contributed to more precise needle placement and reduced incidence of accidental dural puncture. Another possible explanation is that the local anatomical features of each patient were reviewed on MRI prior to the procedure, and anticipating these variations may have reduced the likelihood of subarachnoid migration by allowing a more tailored approach to needle trajectory and positioning. Importantly, visual identification of blood or subarachnoid fluid by aspiration or observation of fluid in the needle hub prior to injection demonstrated low sensitivity. In this study, such findings were present in only 1 of 3 injections with confirmed vascular uptake (33%) and in none of the 4 injections with subarachnoid spread (0%). These results highlight the limited sensitivity of visual identification and reinforce the critical role of fluoroscopic guidance with contrast to ensure safe and accurate needle placement. The findings in our study alight with human guidelines and recommendations, which advocate for the combined use of image guidance, aspiration, and contrast studies to minimise risks and improve procedural safety (55).

The L7 spinal nerve exits through a complex foraminal compartment enclosed by a circumneural sleeve that interfaces with vascular and fascial structures. Minor variations in needle position relative to this sleeve can markedly influence the spread of the injectate, favouring paravertebral, epidural, or subarachnoid spread (19), and increase the risk of inadvertent intravascular injection. In the clinical setting, we addressed this challenge by combining ultrasound and fluoroscopy guidance. Ultrasound enabled real-time soft tissue visualisation, minimising the risk of vascular puncture and allowing optimisation of cannula trajectory. Fluoroscopy was then used to confirm preliminary cannula placement, assess injectate distribution, allowing for fine-tune cannula adjustments in case of accidental intravascular or subarachnoid contrast distribution. This dual-imaging strategy aligns with the ALARA (As Low As Reasonably Achievable) principle of radiation safety (56). In our recent study (57), we demonstrated that the combined use of fluoroscopy and ultrasonography during spinal interventions in dogs results in radiation exposure levels at the lower end of those reported in human medicine for comparable procedures, further supporting the clinical applicability of this approach.

In dogs with lumbosacral stenosis, the epidural space at the lumbosacral junction is often narrowed or distorted due to intervertebral disc herniation, ligamentous hypertrophy, or vertebral remodelling (2–4). These anatomical alterations can lead to displacement or compression of neural structures, increasing the risk of complications when accessing the epidural space during interventional procedures. The traditional interlaminar approach involves advancing a stiff, non-compliant needle from the epaxial region toward the epidural space. In dogs with CDLSS, herniation of the intervertebral disc toward the spinal canal frequently displaces the cauda equina and dural sac dorsally, displacing these structures directly in the trajectory of the advancing needle. This poses patients with moderate to severe lumbosacral stenosis at a higher risk of dural puncture or iatrogenic neural trauma when using the traditional lumbosacral access.

To minimise procedural risks and enable precise drug delivery, we combined sacrococcygeal catheterisation with a foraminal injection technique. This strategy aimed to target pain generators more effectively, particularly in dogs affected with lumbosacral intervertebral disc herniation and radiculopathy, both recognised predictors of lumbosacral pain in dogs (5). The epidural catheter was introduced through the sacrococcygeal space using a Tuohy needle. Although the termination of the dural sac can vary across dogs, it typically ends at or cranial to the sacrum, making sacrococcygeal access a practical approach that minimises the risk of inadvertent dural puncture (58, 59). The compliant and flexible catheter, was then advanced cranially to a position just caudal to the lumbosacral stenosis. This approach avoided passing directly through the stenotic region, which is critical given the significant epidural space narrowing at this site. Such narrowing limits the capacity of neural structures to displace during catheter advancement, increasing the risk of iatrogenic neural injury. However, based on our clinical experience, injection through the catheter alone may not consistently achieve distribution of the injectate to the affected spinal nerve within the stenotic foramina. In addition, even when epidural spread is achieved following foraminal injection, the injectate may not consistently distribute caudally through the stenotic lumbosacral spinal canal toward lumbosacral neural structures. This dual-modality guidance likely enhanced precision of drug delivery to the target structures while reduced procedural risks and complications. Interestingly, the combination of transforaminal and caudal epidural injections in human has shown to achieve greater therapeutic effects in the short- and long- term, when compared with transforaminal epidurals alone (60).

Importantly, the foraminal approach also enabled for the concurrent use of PRF, a technique widely used in medicine for managing radicular pain. In this context, PRF has been shown to enhance the efficacy of transforaminal epidural corticosteroid injections and is considered both safe and effective (25–29). In our study, intraoperative motor stimulation was employed to confirm proximity to the target nerve and minimise the risk of intraneural placement. Although sensory feedback is not feasible in anaesthetised dogs, the combination of imaging guidance and motor stimulation likely provides sufficient accuracy for safe and effective targeting of the DRG.

Following optimal cannula positioning, PRF was delivered using default settings (42 °C, 2 Hz, 20 ms pulse width, 45 V for 2 min), reflecting parameters commonly used in animal models and medicine (61–63). A preclinical canine study using these settings on the sciatic nerve reported no evidence of motor impairment or histopathological damage (64). Although higher voltages and extended application times have been associated with improved outcomes in human patients (65), the safety and efficacy of such modifications in dogs have not yet been established. Future clinical research is needed to define optimal stimulation thresholds and PRF parameters for dogs.

The main limitation of this study is the small sample size, reflecting the available caseload and the application of strict inclusion and exclusion criteria designed to minimise confounding effects from comorbidities that could influence pain and QoL. While this reduced the number of eligible cases, it enhanced internal validity and allowed for a more accurate evaluation of treatment effects in dogs with isolated lumbosacral pain. Cautious interpretation is therefore recommended when extrapolating these findings to patients with coexisting musculoskeletal disorders, in whom a comprehensive assessment of pain sources and potential confounding factors remains essential. The retrospective design and variable follow-up intervals also limit the ability to draw causal inferences. Future larger, randomised, prospective studies would further strengthen outcome evaluation.

In conclusion, image-guided interventional procedures resulted in a long-term clinically relevant improvement in the majority of dogs with lumbosacral pain refractory to conservative management. The addition of PRF was consistently associated with greater benefits across all outcome domains. Although statistical significance was reached only for QoL, the direction and consistency of the effects, along with the absence of adverse events, support the potential value of PRF as a safe adjunctive modality. Given the low sensitivity of visual identification techniques for detecting vascular or subarachnoid needle placement, fluoroscopy with contrast verification is paramount to ensure both efficacy and safety during these procedures. Further prospective, controlled studies are warranted to confirm these findings, refine PRF protocols, and define the role of targeted image-guided interventional techniques in the long-term management of lumbosacral pain in dogs.

## Data Availability

The original contributions presented in the study are included in the article/supplementary material, further inquiries can be directed to the corresponding author.
