# Supplementary material for: Interventional pain management in dogs with lumbosacral stenosis: preliminary long-term clinical outcomes of combined foraminal and epidural injections with or without pulsed radiofrequency
Source: Front Vet Sci. 2026 Jan 12;12:1730491. doi: 10.3389/fvets.2025.1730491 (PMC12833525; doi:10.3389/fvets.2025.1730491)
Supplement: Supplementary file 1 [file Data_Sheet_1.pdf]

Pet's Name:
Case number:
Owner's name: **Address:**

Signalment:

Problem list:

Treatment:

Procedure(s):

I, the undersigned, being the owner of the pet or duly authorised person, give my consent for the above mentioned procedure(s), and any procedure that should become necessary in the interest of the patient.

I hereby give permission for the animal described above to undergo anaesthesia. The method of effective anaesthesia will be determined by the anaesthetist. The use of modern anaesthetic techniques minimises risk however I understand that all anaesthetic procedures carry some element of risk.

There are risks associated with all medical and surgical procedures. The most common complications have been discussed with me and I understand what I have been told. Common complications associated with these procedures include…………………………………

This is not a comprehensive list and other complications can occur.

In order to protect the welfare of my animal, in the unlikely event of an emergency, or where additional pain relief or sedation may be required, I understand the veterinary surgeon may decide to use medicines that are not authorised for use in pets.

I understand that the estimated cost given is not a precise quote. I acknowledge that it can be difficult to accurately estimate cost as additional investigations or treatments may become necessary. I understand that should my estimate be exceeded you will endeavour to contact me. If you are unable to contact me, I authorise you to proceed in the manner which you consider to be in the best interest of my pet. This estimate does not include costs related to re-examinations, follow up treatment or treatment of complications.

I understand that if my account reaches or is forecast to reach £5000, I will be asked to make an interim payment. My remaining account must be settled at the time of my pet's discharge from the hospital, otherwise a surcharge may be levied. I accept that any costs incurred in recovering unpaid fees will be added to my account.

ESTIMATE (*Inclusive* of VAT at 20%) from to

I have read and understood the above and the following page concerning contributions to clinical research. Any questions I had have been answered:

Date: **Signature:**

Contact Telephone Numbers in order of preference;

Does your pet have any metallic implants (other than a microchip)? Y/N

When did your pet last eat?

Has your pet any adverse drug reactions? Y/N

CONSENT BY CLIENT PET OWNERS FOR CONTRIBUTIONS TO CLINICAL RESEARCH IN LINNAEUS BUSINESS UNITS

- I understand that North Downs Specialist Referrals is a business unit of Linnaeus Veterinary Limited (Company no: 10790375), with a registered address at Friars Gate, 1011 Stratford Road, Shirley, West Midlands, B90 4BN, United Kingdom ("Linnaeus"). Linnaeus and any company within the Linnaeus group of companies, which includes:
    - Linnaeus;
    - any subsidiary or holding company from time to time of Linnaeus; and
    - any subsidiary from time to time of a holding company of Linnaeus

    is a part of MARS Petcare ("Group Companies").

- I understand that Linnaeus is committed to providing the highest standards of care to animals and contributing knowledge and expertise to the veterinary community to help improve animal health and welfare.
- I freely give my consent for:

- clinical data archived in my pet’s medical record, diagnostic images (e.g. radiographs, ultrasounds, CT or MRI scans), photographs and videos to be used anonymously for clinical and health research, training and publication purposes;
- any residual samples from those collected for diagnostic purposes (e.g. fluid, faecal or tissue samples) that are not required for testing to be stored for future use in clinical and health research;
- anonymised clinical data, images and archived fluid, faecal or tissue samples to be shared within the Linnaeus Group Companies or with third parties and used in ethically approved clinical research projects undertaken by the Linnaeus Group Companies and their collaborators (whether academic or non-academic). I agree that the Linnaeus Group Companies and their collaborators shall be free to commercialise the results of any such approved projects and that I am not entitled to receive any financial benefit from this.
- Linnaeus, through its business unit, North Downs Specialist Referrals and any of its Group Companies, contacting me (e.g. by telephone, email or by writing) to ask for follow up information on my animal to allow for clinical research and internal auditing of cases; this may be useful if, for example, a specific condition or disease was being evaluated.
